# Photoprotective Effects of Yeast Pulcherrimin

**DOI:** 10.3390/molecules29204873

**Published:** 2024-10-14

**Authors:** Dorota Kregiel, Agnieszka Krajewska, Agnieszka Kowalska-Baron, Karolina H. Czarnecka-Chrebelska, Adriana Nowak

**Affiliations:** 1 Department of Environmental Biotechnology, Lodz University of Technology, Wólczańska 171/173, 90-530 Lodz, Poland; adriana.nowak@p.lodz.pl; 2 Institute of Natural Products and Cosmetics, Faculty of Biotechnology and Food Sciences, Lodz University of Technology, Stefanowskiego 2/22, 90-537 Lodz, Poland; agnieszka.krajewska@p.lodz.pl (A.K.); agnieszka.kowalska-baron@p.lodz.pl (A.K.-B.); 3 Department of Biomedicine and Genetics, Medical University of Lodz, Mazowiecka 5, 92-215 Lodz, Poland; karolina.czarnecka@umed.lodz.pl

**Keywords:** *Metschnikowia pulcherrima*, pulcherrimin, cytotoxicity, HaCaT cells, photoprotection, SPF, UVA/UVB ratio

## Abstract

Sunscreen products can protect the skin against the harmful effects of UV radiation, including reddening, aging, and cancer. The aim of this research was to evaluate the photoprotective effects of yeast pulcherrimin, an iron-chelating dipeptide. We first investigated the cytotoxicity of pulcherrimin produced by *Metschnikowia pulcherrima* yeast on the human keratinocyte HaCaT cell line, using the PrestoBlue assay. We assessed the ability of pulcherrimin to induce DNA repair after the exposure of HaCaT cells to oxidative stress. We also evaluated its protective activity against UVC radiation. The sun protective factor (SPF) was calculated using the Mansur equation. The UVA/UVB ratio values for pure pulcherrimin were evaluated using the Boots Star Rating system. The critical wavelength was determined by calculating the integrated optical density curve area. Based on the results, pulcherrimin shows strong cytoprotective effects through antioxidant and photoprotective activities on the HaCaT cell line. The calculated SPFs were 20 and 15 at pH = 7 and pH = 10, respectively. The critical wavelength above 370 nm and the UVA/UVB ratio R > 1 suggest that yeast pulcherrimin—a cyclic dipeptide containing iron—may be considered a promising photoprotective agent.

## 1. Introduction

Solar emissions include visible light (400–700 nm), heat, and ultraviolet radiation (UVR). Just as visible light consists of different colors that become apparent in a rainbow, the UV radiation spectrum is divided into three regions called UVA (315–400 nm), UVB (280–315 nm), and UVC (100–280 nm). UVB accounts for 5% of the terrestrial UV radiation of sunlight and UVA accounts for 95%. In general, UVC is the most damaging type of UV radiation. However, it is completely filtered by the atmosphere and does not reach the Earth’s surface [1].

UVR present in sunlight is a major environmental factor capable of affecting human health and well-being. It is needed for production of active vitamin D; however, it generates undesirable effects. The organ primarily affected by UVR is the skin, which is composed of a variety of different cell types [2]. The radiation action in the skin depends on its wavelength. UVB radiation penetrates only into the epidermis, but UVA penetrates into the dermis. In turn, visible radiation can reach the subcutaneous adipose tissue [3]. UVC radiation emitted by mercury vapor lamps has been applied for over a hundred years as an effective agent against various microorganisms and viruses. Unfortunately, UVC radiation is also harmful to human cells and tissues.

UVR is primarily recognized for its harmful effects such as autoimmune disorders, carcinogenesis, skin aging, or eye damage. The many mechanisms induced by UVR include rapid or slow activation of the local and central neuro–immuno–endocrine responses involving brain, endocrine, and immune systems, with secondary effects on peripheral organs. These mechanisms may lead to the activation of local neuro–immuno–endocrine and regulatory effects with their projection to the central or systemic level [4]. This invites possible therapeutic UVR applications, for example, in the management of autoimmune and mood disorders, addiction, and obesity [5]. However, UV absorption not only triggers mechanisms that may regulate global homeostasis, but also induces skin pathology, such as erythema, premature cutaneous photoaging, and skin cancer development [3]. Exposure to UVR, meanwhile, is regarded as the major cause of extrinsic skin aging. Excessive exposure of the skin to UV beams triggers a remodeling of the immune system and leads to the photoaging state, which is similar to chronological aging. Repeated long-term exposure to sunlight or UV lamps evokes dynamic alterations in the skin, which are different from those observed during the natural aging process [6,7,8].

Sunlight contains a much greater amount of UVA radiation than UVB radiation. As a result, the relative solar immunosuppressive response is threefold higher for UVA than for UVB. It is worth noting that there is significant interaction between UVB and UVA radiation in sunlight in terms of immunosuppression in human skin. UVB light induces an earlier intensive response, whereas UVA has a slower effect [7]. The important targets of UV beams are nuclear and mitochondrial DNA. UVR is able to damage DNA either directly, by forming pyrimidine dimers, or indirectly, by stimulating oxidative stress, which oxidizes guanine bases generating 8-oxo-7,8-dihydroguanine (8-oxoG). Modification of 8-oxoG is a hallmark of aging tissues and age-related diseases. Interestingly, DNA damage is a potent inducer of inflammatory responses via different pathways. Given that inflammation can also trigger DNA damage, it seems that UVR exposure can create a vicious cycle which is a possible source of the photoaging process in the skin. UVR also stimulates the degradation of dermal components of the extracellular matrix, such as collagen, elastin, and glycoproteins. Similar changes occur in natural aging processes, but in the case of UVR exposure they occur much faster [8]. The progression of skin photoaging and cancer development is closely linked to the overproduction of dermal inflammatory cells—monocytes and epidermal keratinocytes [7]. Keratinocytes undergo apoptosis due to irreversible and severe DNA damage caused by UV exposure.

Photoprotective care products often contain UV filters, which have the ability to absorb, scatter, or reflect UV radiation. A full list of UV filters permitted for use in cosmetics is provided in Annex VI of Regulation WE 1223/2009 [9]. UV filters are divided into organic (chemical) and mineral filters. Chemical filters include UVA filters (e.g., dibenzoylmethane derivatives), UVB filters (e.g., derivatives of p-aminobenzoic acid, p-methoxycinnamic acid, salicylic acid, and camphor), and a wide range of both UVA and UVB filters (e.g., benzophenones and phenylbenzotriazoles). Mineral filters are pigments with particle sizes of 200–300 μm or below 100 μm (e.g., titanium dioxide, zinc oxide). Mineral UV absorbers usually form a whitish layer on the skin that effectively scatters light [10]. However, the whitish hue and white residue of care products may not be desirable, especially for people with dark skin.

According to the latest knowledge, effective protectants should contain both antioxidants (which have additional benefits against UV radiation) and anti-aging agents. Products that are easy to apply and do not have a whitish hue may provide better comfort for everyday skin protection [10,11]. Therefore, there is a demand for better alternatives to conventional UV absorbers that provide efficient photoprotection.

Recently, iron compounds have been shown to provide effective protection against UVR, especially red (Fe_2_O_3_), yellow (Fe(OH)_3_/FeOOH), and black (Fe_3_O_4_) iron oxides. Sunscreens formulated with iron preparations have been applied for the treatment of melasma and the prevention of hyper-pigmentation [10,11,12]. The use of iron agents in skin care products is not novel. However, their use in different formulations is a relatively new development. Recent studies have demonstrated that iron chelators can be used in environmental or atmospheric aging protection, including sunscreens [13,14]. Therefore, iron chelators that prevent or inhibit the toxic effects of UVR may offer a simple approach to preventing skin aging [15,16]. In this context, low molecular peptides have recently attracted particular interest, due to their superior bioactivity, solubility, hydrophilicity, absorption, and stability in comparison to conventional UV filters [17]. A number of peptides with iron-chelating abilities have been identified, including the enzymatic hydrolysates of dietary proteins from whey, milk, cottonseed meal, porcine blood plasma, fish collagen, soybean, and spirulina [17,18]. However, less attention has been given to peptides of microbial origin.

An interesting alternative may be pulcherriminic acid. It is a product of fermentation processes by some yeasts from the genera *Metschnikowia*, *Lipomyces*, *Kluyveromyces*, and *Dipodascopsis*, as well as by some bacteria belonging to the *Bacillus* genus [19,20,21,22]. Chemically, pulcherriminic acid is a dipeptide (L-Leu-L-Leu) that consists of two leucine amino acids linked together in a cyclic form. The molecular formula and molecular weight of pulcherriminic acid are C_12_H_20_N_2_O_4_ and 256.2980 g/mol, respectively. It is soluble in water. Pulcherriminic acid is known to have the ability to chelate Fe^3+^, producing reddish-brown pulcherrimin [20,21,22]. The molecular formula of pulcherrimin is C_12_H_18_N_2_O_4_Fe_2_, with maximum UV absorption peaks at three different wavelengths: 240, 280, and 410 nm. Pulcherrimin is almost insoluble in water and organic solvents such as ethanol, but it can be easy dissolved in NaOH solution [23]. Pulcherrimin is an effective agent with important ecological roles. This iron chelate has been reported to have antioxidative and antibiofilm properties. It also shows significant hydrophilic characteristics and photoprotection against yeast cell death [24].

Here, we investigate the photoprotective effects of pulcherrimin on human skin. We evaluate the cytoprotective properties of pulcherrimin for human HaCaT skin keratinocytes, as well as its antioxidant and photoprotective properties against UV radiation. This is the first paper to describe these unique properties of pulcherrimin of yeast origin.

## 2. Results and Discussion

### 2.1. Cytotoxicity of Pulcherrimin against HaCaT Cells

**Figure 1:**
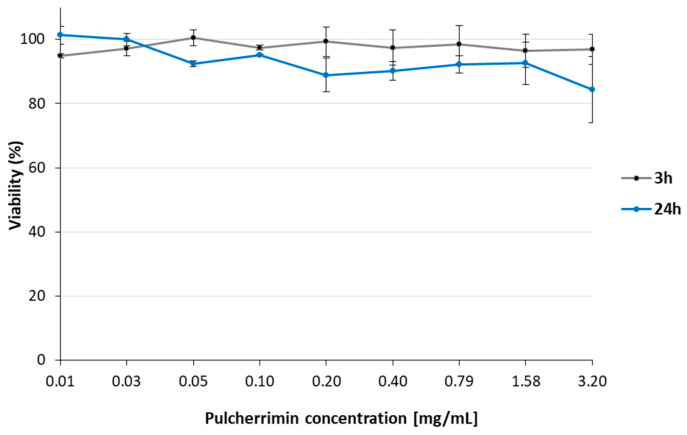
Viability of human skin keratinocyte HaCaT cells determined by the PrestoBlue assay after 3 h and 24 h of exposure to pulcherrimin. Each value represents the mean of four or five repeats ± the standard deviation (SD). The results are from two independent experiments. Results not statistically significant from the negative control, which represented 100% viability (ANOVA, *p* ≤ 0.05).

In the first stage of the study, the cytotoxicity pulcherrimin was evaluated against the HaCaT cell line using the PrestoBlue assay. The HaCaT cells were treated with nine concentrations of pulcherrimin, and cytotoxicity was evaluated at two time points: after 3 h and after 24 h of exposure. The results are presented as the mean cell viability [%] ± standard deviation (SD) from two independent experiments (Figure 1). After 3 h of exposure to pulcherrimin, HaCaT cell viability was very high, ranging from 94.83% ± 0.71% to 100.46% ± 2.39% depending on the concentration. After 24 h of exposure, the HaCaT cells also showed high viability of 90.18% ± 2.92% to 101.31% ± 2.80% over a concentration range of 0.01–1.58 mg/mL. Only in the presence of the highest tested concentration of pulcherrimin, i.e., 3.20 mg/mL, did cell viability decrease slightly, to 84.40% ± 10.30%. Since the IC_50_ (half-maximal inhibitory concentration) value cannot be determined, pulcherrimin can be considered (at the concentration and time tested) to be non-cytotoxic.

From the results, it can be concluded that at the tested concentrations of pulcherrimin did not show cytotoxicity towards HaCaT cells after 3 h or after 24 h of exposure. This lack of cytotoxicity confirmed that pulcherrimin was a promising candidate for further research into its potential for use in skin protective applications.

In our previous studies, we tested the cytotoxicity of the same concentrations of pulcherrimin against five different cell lines: Caco-2 (human colon adenocarcinoma), Hep-G2 (human hepatocellular carcinoma), HeLa (human cervical adenocarcinoma), A-549 (human lung alveolar adenocarcinoma), and IEC-6 (rat normal small intestine) [25]. The cells were exposed to pulcherrimin for 48 h. Pulcherrimin proved highly cytotoxic to almost all the tested cell lines. Caco-2 and IEC-6 cells were the most sensitive to pulcherrimin, followed by HeLa, then HepG-2. The least sensitive cell line was A-549. Since pulcherrimin is not soluble in aqueous solutions, but only in alkaline solutions, we previously studied, as in the present research, a suspension of pulcherrimin in a cell culture medium. We concluded that the cytotoxic effect of pulcherrimin is probably due to the physical lesions caused by the suspended pulcherrimin particles. However, in the present study on the HaCaT cell line, we did not observe this effect, possibly due to the shorter exposure time of the cells, i.e., 3 h and 24 h. To date, the cytotoxicity of bacterial pulcherrimin (from *Bacillus subtilis*) has been tested against normal fibroblast-like kidney Vero cells and cancer HepG-2 cells, demonstrating strong cytotoxicity in the range of similar concentrations of 0.15 to 5 mg/mL [26].

### 2.2. Protective Activity of Pulcherrimin after Subjecting HaCaT Cells to Oxidative Stress

**Figure 2:**
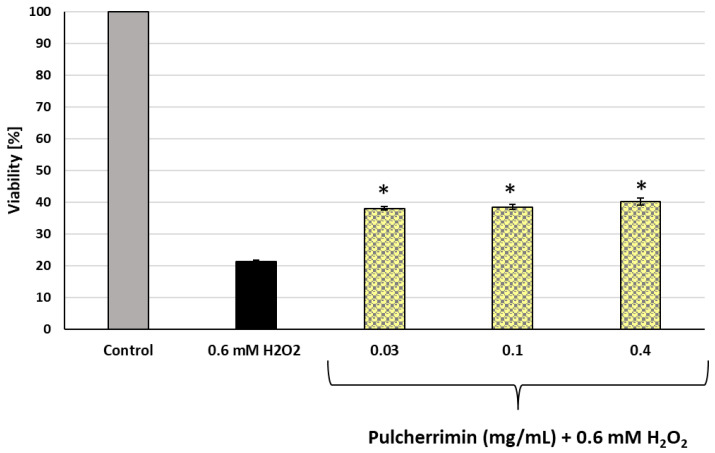
Activity of pulcherrimin against oxidative stress in human skin keratinocyte HaCaT cells. HaCaT cells were first exposed to non-toxic (IC_0_) concentrations of pulcherrimin for 24 h, then incubated for 4 h in the presence of 0.6 mM H_2_O_2_. The viability of HaCaT cells was measured using the PrestoBlue assay. Each value represents the mean of eight repeats ± the standard deviation (SD). Asterisks indicate results are statistically significant from 0.6 mM H_2_O_2_ (ANOVA, *p* ≤ 0.05).

The cytoprotective activity of pulcherrimin was assessed against H_2_O_2_-induced oxidative stress. Cell viability was evaluated using the PrestoBlue assay. Figure 2 shows that pre-incubation of HaCaT cells with pulcherrimin for 24 h at the three non-cytotoxic concentrations (IC_0_) significantly (*p* ≤ 0.05) increased cell viability compared to the positive control (cells exposed to H_2_O_2_ without pre-incubation with pulcherrimin). Cell viability after pre-incubation with pulcherrimin was 2-fold higher than without pulcherrimin and was not dependent on the concentration of pulcherrimin. For the positive control (0.6 mM hydrogen peroxide), the average cell viability was 21.34% ± 0.42%, while with pulcherrimin, it ranged from 38.11% ± 0.49% to 40.22% ± 1.14 for its concentrations of 0.03 and 0.4 mg/mL, respectively. All results were statistically significant from the negative control (*p* ≤ 0.05).

**Figure 3:**
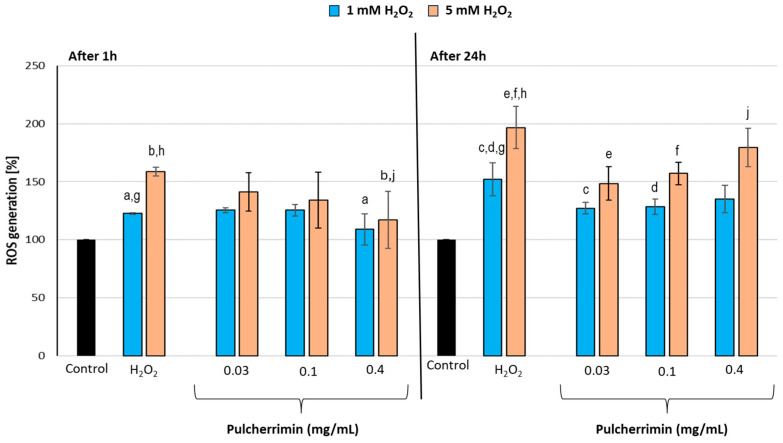
Effect of pulcherrimin on ROS generation in human skin keratinocyte HaCaT cells. The cells were first pre-incubated with non-toxic (IC_0_) concentrations of pulcherrimin for 1 h or 24 h, then exposed to 1 mM and 5 mM H_2_O_2_ for 30 min. Each value represents the mean of the fluorescence values from 3–6 individual wells ± the standard error of the mean (S.E.M.). The same letters indicate results are statistically significant; no letter means no statistical significance (ANOVA, *p* ≤ 0.05).

Next, we studied the effect of pulcherrimin on the induction of reactive oxygen species (ROS) in HaCaT cells (Figure 3). After 1 h pre-incubation with pulcherrimin, the cells showed inhibited ROS generation in response to oxidative stress induced by both 1 mM and 5 mM of H_2_O_2_. The strongest statistically significant (*p* ≤ 0.05) antioxidant activity was exhibited by the highest tested concentration of pulcherrimin (0.4 mg/mL), which inhibited ROS induction by 11% and 26% for H_2_O_2_ concentrations of 1 mM and 5 mM, respectively. After 24 h pre-incubation of cells with pulcherrimin, about 23% higher intracellular ROS generation was observed than after 1 h pre-incubation in both concentrations of H_2_O_2_ tested (*p* ≤ 0.05). After 24 h pre-incubation with pulcherrimin, cells incubated with 1 mM and 5 mM H_2_O_2_ also showed inhibited ROS generation. Pulcherrimin concentrations of 0.03 mg/mL and 0.1 mg/mL inhibited ROS induction by about 16% for 1 mM H_2_O_2_ (*p* ≤ 0.05). With 5 mM H_2_O_2_, ROS induction was inhibited by 20% and 25% for pulcherrimin concentrations of 0.1 mg/mL and 0.03 mg/mL, respectively (*p* ≤ 0.05). The highest tested concentration of pulcherrimin (0.4 mg/mL) inhibited the induction of ROS by H_2_O_2_ the least, by 11% for 1 mM and 9% for 5 mM H_2_O_2._ These results were not statistically significant. In previous experiments, we showed that pulcherrimin can induce ROS and H_2_O_2_ release in Caco-2 cells after 48 h of cell exposure, starting at a concentration of 0.1 mg/mL [25]. This also explains why the cytoprotective effect of pulcherrimin observed in the present study at a higher concentration, i.e., 0.1 mg/mL, is weaker after 24 h of cell exposure. Jayalakshmi et al. (2012) demonstrated that pulcherrimin from *B. subtilis* displayed antioxidant activity in 2,2-diphenyl-1-picrylhydrazyl (DPPH) assay [26].

**Figure 4:**
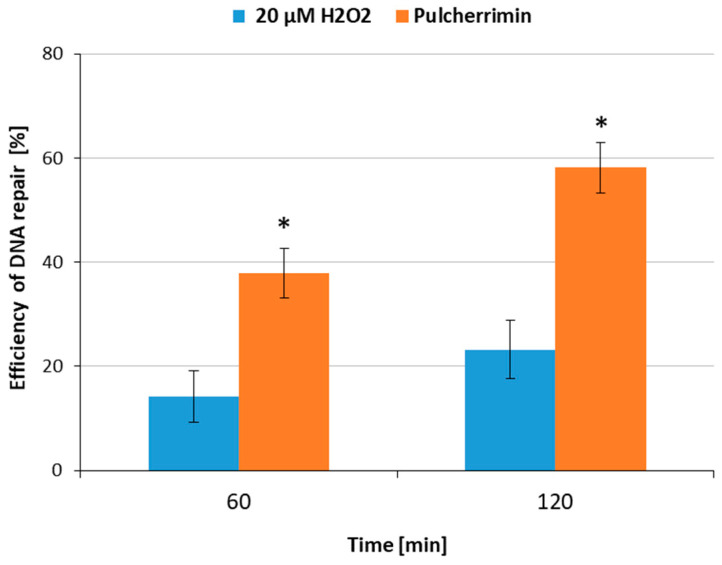
DNA repair efficiency (%) in human skin keratinocyte HaCaT cells exposed for 10 min to mutagen H_2_O_2_ (20 µM) on ice. The cells were post-incubated (at 37 °C for 60 min and 120 min) with 0.4 mg/mL pulcherrimin and DNA repair was measured at time intervals. For each time interval, 50 cells were analyzed. Error bars denote the standard error of the mean (S.E.M.). Asterisks indicate results are statistically significant from the positive control (H_2_O_2_) at each time point (ANOVA, *p* ≤ 0.05).

The next stage of the study tested the ability of pulcherrimin to induce DNA repair after the exposure of HaCaT cells to H_2_O_2_-induced oxidative stress, as estimated by the comet assay. The study was conducted for a selected single concentration of pulcherrimin of 0.4 mg/mL, and the results are presented in Figure 4. The rate of DNA damage in the negative control at different time points ranged from 0.82% ± 0.18% to 5.60% ± 0.52%. It was observed that pulcherrimin significantly increased DNA repair efficiency in the cells after 60 min and 120 min of exposure (*p* ≤ 0.05). The rate of repairs was increased 2.5–2.7-fold in the presence of the tested concentration of pulcherrimin (0.4 mg/mL). These results indicate that pulcherrimin had a cytoprotective effect on H_2_O_2_-induced oxidative stress. In the 2,2-diphenyl-1-picrylhydrazyl (DPPH) assay, pulcherrimin isolated from *Bacillus subtilis* was shown to have antioxidant properties [26].

### 2.3. Protective Activity of Pulcherrimin against UVC Radiation

**Figure 5:**
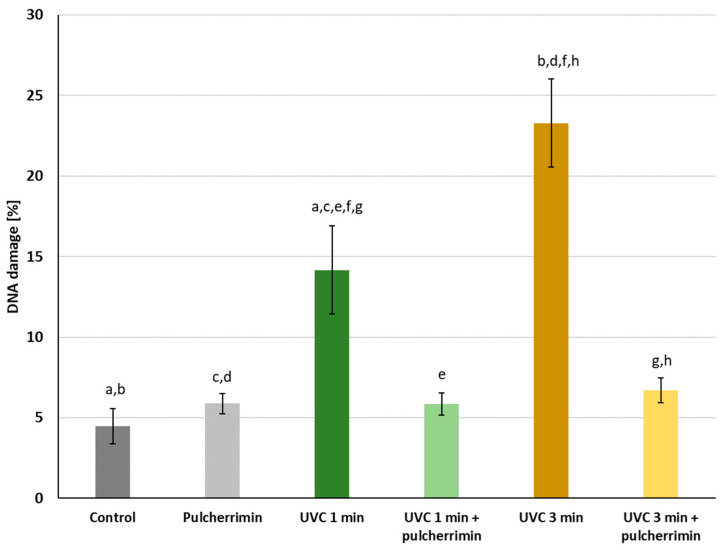
Cytoprotective activity of pulcherrimin against human skin keratinocyte HaCaT cells after UVC irradiation. The cells were pre-incubated (at 37 °C for 60 min) with 3.2 mg/mL of pulcherrimin and then irradiated with UVC for 1 min and 3 min. DNA damage is expressed as the mean DNA content in the tail of the comets ± the standard error of the mean (S.E.M.) in the alkaline comet assay. The number of cells analyzed was 50. The same letters indicate results are statistically significant (ANOVA, *p* ≤ 0.05).

**Figure 6:**
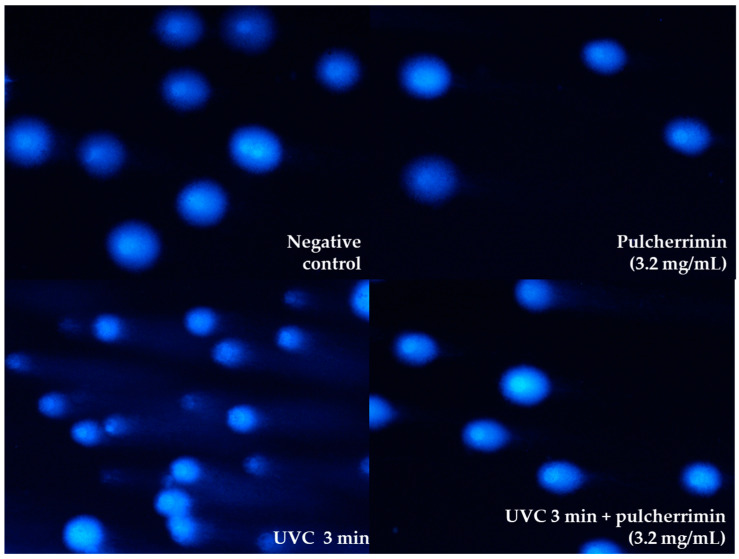
Randomly selected images of DAPI-stained comets exposed to 3.2 mg/mL pulcherrimin and UVC radiation (Nikon Eclipse Ci H600L, Tokyo, Japan), 20× objective.

To investigate whether pulcherrimin exhibits protective activity against UVC radiation, HaCaT cells were pre-incubated for 60 min with a suspension of pulcherrimin at an IC_0_ concentration of 3.2 mg/mL. The cell monolayer was then irradiated with UVC-type radiation for 1 min and 3 min in the presence of pulcherrimin. The results are presented in Figure 5. As can be seen, UVC-induced DNA damage was several times lower in the presence of pulcherrimin than without pulcherrimin. The percentage of DNA damage in the negative control was 4.46% ± 1.1%. In the presence of the tested concentration of pulcherrimin, the percentage of DNA damage was 5.87% ± 0.64%. After irradiation of HaCaT cells with UVC radiation for 1 min, the genotoxicity was 14.17% ± 2.74%. In the presence of pulcherrimin, genotoxicity was only 5.85% ± 0.70%, so DNA damage was 2.5 times lower (*p* ≤ 0.05). After irradiating the HaCaT cells for 3 min, genotoxicity increased to 23.29% ± 2.74%. However, it was 6.71% ± 0.77% in the presence of pulcherrimin, and UVC-induced DNA damage was 3.5 times (*p* ≤ 0.05) lower. It can be inferred that pulcherrimin demonstrates overall a strong cytoprotective effect against UVC radiation on the model skin cell line HaCaT. After longer exposure of the skin cells (3 min), the cytoprotective effect of pulcherrimin against UVC radiation was 1.5 times stronger than with shorter exposure (1 min). Sample images are presented in Figure 6.

In a previous study, Kregiel and co-workers observed that the survival of *M. pulcherrima* cells after UVC treatment was dependent on pulcherrimin productivity [24]. Cell durability after UVC action was the highest (21%) for the strain that was the best producer of pulcherrimin (198 mg/L). The other yeast strains, producing smaller amounts of pulcherrimin, exhibited lower cell survival after UVC treatment. We also confirmed that yeast pulcherrimin—a cyclic dipeptide with iron—provides significant photoprotection against UV-induced damage and cell death [27,28,29].

The presented results are pioneering, previously unpublished, and therefore require refinement and extension. They may be a valuable research approach that can be used to advance the methodology for cell line studies. Since the experiments were performed on immortalized HaCaT skin keratinocytes (with *p53* mutation), the validation of the hypothesis would require testing with the application of primary human keratinocytes and human skin histoculture. This will be the subject of future research.

### 2.4. Determination of SPF for Yeast Pulcherrimin

The interesting results showing the safety of yeast pulcherrimin on the HaCaT cell line and its antioxidant activity gave impetus to further studies, including determination of SPF. The most traditional and officially accepted method for determining SPF is to conduct an in vivo study involving between 10 and 20 human volunteers of both sexes with various skin types. However, this method is expensive and introduces ethical considerations. Therefore, new in vitro techniques have been developed for assessing the efficiency of sunscreen products. These in vitro approaches are generally of two types: (1) measurements of absorption or the transmission of UV radiation through films of sunscreen products on quartz plates or membranes; or (2) methods based on spectrophotometric measurements of diluted solutions of sunscreens [30]. In our research, we used an in vitro method based on spectrophotometric measurements. The SPF values for pulcherrimin solutions were evaluated spectrophotometrically and the Mansur Equation (1) was applied.

**Figure 7:**
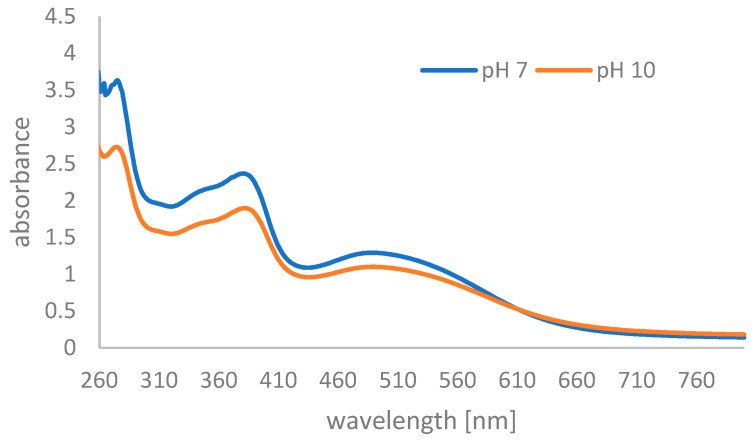
UV-Vis absorption spectra of yeast pulcherrimin at pH = 7 and pH = 10.

In the first step, we evaluated the absorption spectra of pure pulcherrimin at pH 7 and pH 10 (Figure 7). These pH levels were chosen because pulcherrimin is only slightly soluble in neutral environments and much more soluble in alkaline environments than in acidic conditions. Pulcherrimin absorbs in the range of 290–400 nm, so was expected to be effective at preventing the harmful effects of sunlight [23]. Two absorption bands with maxima located at ~390 nm and ~500 nm were revealed in the spectra. The longest wavelength absorption band may be due to the presence of iron in the pulcherrimin molecule. However, the pulcherrimin solutions exhibited the strongest absorption in the 260–300 nm wavelength range (UVC-UVB).

Table 1 shows the results for absorbance and SPFs obtained in four independent experiments. The average SPF values were 20 and 15 at pH = 7 and pH = 10, respectively.

The comparison of the pulcherrimin absorbance at pH = 7 and pH = 10 revealed statistically significant higher absorbance at pH = 7 for all analyzed wavelengths (Mann–Whitney U Test). In addition, the comparison of the pulcherrimin SPF at pH = 7 and pH = 10 revealed statistically significant higher SPF at pH = 7 (Mann–Whitney U Test, *p* = 0.030).

Other results of the statistical analysis are presented in the Supplementary Material (https://www.mdpi.com/article/10.3390/molecules29204873/s1, Table S1: The pulcherrimin absorbance (Mean ± SD) depending on the wavelength. Statistically significant differences according the Kruskal-Wallis (KW) test; Figure S1: The pulcherrimin absorbance (Mean ± SD) at pH = 7 depending on the wavelength; Figure S2: The pulcherrimin absorbance (Mean ± SD) at pH = 10 depending on the wavelength; Figure S3: The pulcherrimin SPF (Mean ± SD) depending on the pH).

Given that pulcherrimin is soluble only in an alkaline environment and only slightly soluble in a neutral pH, it can be assumed that the SPF value for pulcherrimin may be higher in an acidic environment, in which this compound is insoluble. It is worth noting that a substance with an SPF of at least 6 exhibits meaningful photoprotective action [30,31,32]. Therefore, yeast pulcherrimin can be considered as a promising photoprotective agent against UVR, regardless of its degree of solubility.

In vitro testing can be used as a formulation tool to identify new filters, optimize combinations, and pre-screen protective formulas prior to in vivo testing in humans [31]. Since Mansur and coworkers developed a very simple mathematical equation for estimating the sun protection factor in vitro using UV spectrophotometry, this method has been used to determine the SPFs of commercially available sunscreen cosmetics, as well as potential cosmetic ingredients with photoprotective potential [32,33,34,35,36,37,38,39]. The Mansur methodology is routinely used to determine the SPFs of commercially available sunscreens [40]. Various studies have shown that the calculated SPFs are consistent with moderate SPF values declared on packaging, while cosmetics with high declared SPF values (e.g., SPF 50) show a larger deviation from the spectrophotometrically determined values. Therefore, Yang et al. proposed a modified version of the Mansur equation with a different correction factor, for products with a high SPF value [41]. In general, the spectroscopic method based on the Mansur equation is considered to be reliable for determining the SPF of cosmetic ingredients intended as potential UV filters. This method can give useful information on sun protection parameters before proceeding to in vivo tests.

### 2.5. Estimation of the Degree of Protection against UVA

The Boots company has proposed a widely used system of rating sunscreens based on classes. The Boots star system gives sunscreens 3–5 stars, which indicate how much protection the sunscreen offers in the UVA spectrum. Five stars on the Boots system means that UVA protection achieves more than 90% UVB protection [42]. In our study, the determined critical wavelengths for pulcherrimin at pH 7 and pH 10 are above 370 nm, at 387.99 ± 0.47 and 389.00 ± 0.88 nm, respectively (Table 2). According to the Boots system, the R coefficients for yeast pulcherrimin at pH 7 and pH 10 are 1.08 ± 0.02 and 1.05 ± 0.02, respectively (Table 2). This means that yeast pulcherrimin earns 5 stars on the Boots rating system, which is the highest photoprotection rating [43].

Recently, there has been increased interest in research on natural substances with photoprotective properties. A new UV-protecting system with a maximum SPF of 15 has been proposed, based on liposomes/polyhydroxybutyrate with encapsulated coffee extracts [44]. Ethanolic extracts with high SPF values (31.0 and 30.0), λ_cr_ (393.98 and 337.81 nm), and UVA/UVB ratios (1.5 and 1.2) have been developed from *Sloanea chocoana* and *S. pittieriana* plants [45]. Remarkable results have also been reported for the methanolic extract of *Baccharis antioquensis*, an endemic plant species from the high mountain ecosystems of Colombia that demonstrates significant protection against both UVA and UVB radiation with an SPF value of 15 [46]. Micro- and macro-organisms, including molds [47,48], cyanobacteria [49], algae [50,51,52], diatoms [53], and aquatic animals (e.g., sponges [54] and fishes [55]), produce active compounds with UVR absorption capacity. These active compounds include mycosporins and mycosporine-like amino acids, fucoidans, and pigments which protect them against high levels of UVR. Other UV-protective natural resources include pigmented yeasts [56,57]. Therefore, the use of yeast pulcherrimin—a cyclic dipeptide containing iron—fits perfectly into the search for new natural sun protectors. Our results suggest that this iron chelate could provide effective skin protection against UV light. While these findings are promising, further research is necessary to fully explore potential applications of yeast pulcherrimin in skincare products.

## 3. Materials and Methods

### 3.1. Chemicals and Other Materials

Dulbecco’s Modified Eagle’s Medium (DMEM), phosphate buffer saline (PBS) for cell cultures, 4-(2-hydroxyethyl)-1-piperazineethanesulphonic acid (HEPES), streptomycin–penicillin mixture for cell cultures, 2′,7′-dichlorofluorescin diacetate (DCFH–DA), trypan blue, hydrogen peroxide (H_2_O_2_), LMP (low melting point) and NMP (normal melting point) agaroses, sodium chloride (NaCl), Triton X-100, EDTA, Tris, 4′,6-diamidino-2-phenylindole (DAPI), and sodium hydroxide (NaOH) were purchased from Merck Life Science, Warsaw, Poland. Fetal bovine serum (FBS), GlutaMAXTM, TrypLETM Express, PrestoBlue, and roux flasks were purchased from Thermo Fisher Scientific, Waltham, MA, USA. In addition, 6- and 96-well plates and serological pipettes (Greiner Bio-One GmbH Kremsmünster, Austria) were purchased from Biokom Systems, Janki, Poland. Ready-to-use 0.01 M PBS with pH 7.4 was purchased from S.WITKO CHS, Łódź, Poland. Borate buffer was purchased from Chempur, Piekary Śląskie, Poland.

### 3.2. Obtaining Yeast Pulcherrimin

Pulcherrimin was obtained from the yeast strain *Metschnikowia* sp. LOCK 1144 cultivated in minimal broth [1% glucose (*w*/*v*), 0.3% (NH_4_)_2_SO_4_ (*w*/*v*), 0.1% KH_2_PO4 (*w*/*v*), 0.05% MgSO_4_ × 7H_2_O (*w*/*v*), 0.1% yeast extract (*w*/*v*), 0.1% casein-peptone (*w*/*v*), 0.05% FeCl_3_ (*w*/*v*)] after 72 h incubation on a rotary shaker (230 rpm) at 25 °C. Pulcherrimin was extracted with methanol (50 mL of 99.8% methanol per 10 g of wet yeast biomass) at 4 °C, then purified by dissolution in 2N NaOH. Precipitation in HCl was repeated three times. Finally, the red pigment obtained was collected by centrifugation. Quantitative determination of pulcherrimin was conducted spectrophotometrically at the maximum absorption wavelength of 410 nm, according to our previous study [23]. The stock concentration of pulcherrimin (suspended in cell culture medium) was 31.65 mg/mL, and stored at −20 °C until analysis.

### 3.3. Cell Cultures

The HaCaT cell line (normal immortalized human keratinocyte) from the 35th passage (original material created by Prof. Dr. Petra Boukamp and Dr. Norbert Fusenig) [58] was used. In brief, the cells (Cell Line Service GmbH, Eppelheim, Germany) were cultured in DMEM with the addition of GlutaMAX^TM^ (2 mM), HEPES (25 mM), FBS (10%), and streptomycin/100 IU/mL with penicillin (100 μg/mL) mix at 37 °C in 5% CO_2_ atmosphere. The cells were detached with TrypLE^TM^ Express and centrifuged (182× *g*, 3 min). Viability was determined with trypan blue and had to be at least 90%.

### 3.4. PrestoBlue Assay

Pulcherrimin cytotoxicity was assessed with PrestoBlue reagent. A total of 10,000 cells/well were seeded into a black 96-well plate and incubated for 24 h at 37 °C (95% CO_2_). The next day, the medium was aspirated and dilutions of the pulcherrimin suspension in culture growth medium were added in final concentrations from 0.01 to 3.2 (mg/mL) in 4–5 replicates. The cells were exposed to pulcherrimin for 3 h and 24 h (37 °C, 5% CO_2_). The negative control was cells in culture medium. The positive control was dilutions of 10% dimethyl sulfoxide (results not published). The pulcherrimin was removed. PrestoBlue (10% solution in PBS) was added to each well. The cells were incubated for a further 2 h (37 °C, 5% CO_2_). The fluorescence was measured in a microplate reader at λ_ex_ 560 nm and λ_em_ 590 nm. The fluorescence of untreated cells represented 100% viability.

### 3.5. Antioxidant Activity of Pulcherrimin

#### 3.5.1. Measurement of Cell Viability after Exposure to H_2_O_2_ with Pulcherrimin Pre-Incubation

First, 10,000 cells were loaded into each well in a black 96-well plate and incubated for 24 h at 37 °C (95% CO_2_). Next, the medium was changed for fresh and pulcherrimin at non-toxic (IC_0_) concentrations (0.03; 0.1; 0.4 mg/mL) was added on the cell monolayers (in 8 replicates). The samples were incubated (24 h, 37 °C, 95% CO_2_). The negative control was cells without pulcherrimin. The next day, the pulcherrimin was removed, the cells were double washed with PBS, and H_2_O_2_ in cell culture medium without supplements was added at final concentrations of 0.2 mM, 0.4 mM, and 0.6 mM. The positive control consisted of cells incubated only with the tested concentrations of H_2_O_2_ (without pre-incubation with pulcherrimin). The samples were incubated for a further 4 h under the same conditions. The H_2_O_2_ was removed and the cells were washed with PBS. The PrestoBlue assay was then performed, according to the procedure described in Section 2.4.

#### 3.5.2. Measurement of ROS after Exposure to H_2_O_2_ and Pre-Incubation with Pulcherrimin

First, 10,000 cells were loaded into each well in a black 96-well plate and incubated for 24 h at 37 °C (95% CO_2_). Next, the medium was changed for fresh and pulcherrimin at non-toxic (IC_0_) concentrations (0.03; 0.1; 0.4 mg/mL) was added on the cell monolayers (in 3–6 replicates). The samples were then incubated for 1 h and 24 h (37 °C, 95% CO_2_). The negative control was cells without pulcherrimin. The next day, the pulcherrimin was removed, the cells were double washed with PBS, and DCFH–DA (20 µM) was added to each well along with culture media without FBS and incubated for 30 min (37 °C, 5% CO_2_) in the dark. Subsequently, the DCFH–DA was removed, the cells were double washed with PBS, and H_2_O_2_ in cell culture medium without supplements was added at final concentrations of 1 mM and 5 mM. The cells were incubated for a further 30 min. The positive control consisted of cells incubated only with the tested concentrations of H_2_O_2_ (without pre-incubation with pulcherrimin). After incubation, the fluorescence was measured (λ_ex_ 490 nm and λ_em_ 530 nm). The average DCF fluorescence was determined as a percentage (%) relative to that of untreated cells, which was assumed to be 100%.

#### 3.5.3. DNA Repair Measurement

A suspension containing 10^5^ HaCaT cells was exposed to 20 µM H_2_O_2_ for 10 min on ice to induce oxidative stress. After centrifugation (182× *g*, 4 °C, 15 min), the cells were exposed for 60 min and 120 min to pulcherrimin at IC_0_ (0.4 mg/mL). To stop DNA repair in the cells, the samples were placed in an ice bath. At each time interval, DNA repair was quantified by determining the extent of residual DNA damage in the comet assay. The positive control was exposed to H_2_O_2_ only. The negative control consisted of cells in DMEM. The alkaline comet assay was performed as described in Section 3.7.

### 3.6. DNA Damage Induced by UVC Radiation

Cells were seeded into 6-well plates at 10^6^ cells/well and cultured for 24 h at 37 °C with 5% CO_2_. Next, the cells were pre-incubated for 60 min with pulcherrimin suspension at an IC_0_ concentration (i.e., not cytotoxic to the cells) of 3.2 mg/mL. Subsequently, in the presence of pulcherrimin, the cell monolayer was exposed to UVC radiation using a UVC lamp (HNS T8 30W G13, Osram GmbH, Germany), at λ = 254 nm and 7800 cd luminous intensity (UVC irradiance 0.24 W/m^2^) for 1 min and 3 min at a distance of 25 cm at room temperature (22 °C). Negative control samples were cells without pulcherrimin and with pulcherrimin added at the above dose and not irradiated with UVC. Positive controls were cells without pulcherrimin, irradiated with UVC for 1 min and 3 min under the same conditions.

### 3.7. Single Cell Gel Electrophoresis Assay (SCGE)

To estimate genotoxicity after UVC irradiation as well as DNA repair, the alkaline version of the comet assay was used to detect single breaks in DNA. Samples were centrifuged for 15 min at 4 °C, 182× *g*, after which the supernatant was pelleted. Agarose with a melting point of 37 °C was added. The supernatant was then applied to warm, double-layered agarose-coated slides and covered with coverslips. The samples were transferred to a Chilling Plate for Comet Assay Slides (Cleaver Scientific, 41 Somers Rd, Rugby CV22 7DH, UK) and allowed to solidify. Once the samples had solidified, the coverslips were removed and alkaline lysis was performed to release the DNA from the cells. The slides were placed in a cuvette, flooded with lysis buffer (2.5 M NaCl, 1% Triton X-100, 100 mM EDTA, 10 mM Tris, pH 10), and incubated for 60 min at 4 °C. After lysis, the buffer was removed. The samples were flooded with developing buffer (300 mM NaOH, 1 mM EDTA) and left for 20 min at 4 °C. They were then washed with electrophoretic buffer (300 mM NaOH, 1 mM EDTA, pH > 13) and placed in an electrophoresis apparatus into which electrophoretic buffer was poured. Electrophoresis was carried out for 20 min at 21 V and 29 mA. The entire analysis was conducted in the dark to avoid additional DNA damage. After electrophoresis, the slides were neutralized in distilled water and left to dry overnight. To determine the extent of DNA damage, slides were stained with the fluorescent dye DAPI at a concentration of 1 µg/mL. Samples were incubated at 4 °C for 60 min in the dark, followed by comet analysis at 200× magnification in a fluorescence microscope (Nikon Eclipse Ci H600L, Japan) equipped with a camera. The Lucia Comet v.7.0 computer program was used to process the test results. For each sample, 50 randomly selected comets were analyzed on the basis of a parameter indicating the percentage of DNA content in the comet’s tail.

### 3.8. In Vitro Spectrophotometric Determination of SPF for Pulcherrimin

SPF was determined according to the method described by Dutra et al. [59], with some modifications. Pulcherrimin solution (3 mg/mL) was added (0.3 mL) to volumetric flasks with a capacity of 5 mL and made up to the line with buffer. Two buffers were used: 0.01 M PBS (S.Witko, Łódź, Poland) with pH 7 and borate (Chempur, Piekary Śląskie, Poland) with pH 10. The concentration of pulcherrimin in the samples was therefore approximately 0.2 mg/mL. The contents of the 5 mL flasks were exposed to ultrasound at a frequency of 25 kHz for 30 min, and then filtered through Whatman 1540–125 filter paper.

The absorption spectra were recorded using a Nicolet Evolution 300 double-beam spectrophotometer (Thermo Electron Corporation, Cambridge, UK) equipped with a 150 W xenon lamp. All measurements were performed in quartz cuvettes at 20 °C. SPF values were calculated using the Mansur equation (Equation (1)) [34]:

(1) $$SPF=CF\cdot \sum_{290}^{320}EE\left(\lambda \right)\cdot I\left(\lambda \right)\cdot A\left(\lambda \right)$$

where CF (=10) is the correction factor estimated in such a way that a standard 8% Homosolate filter solution had a SPF value of 4 determined by UV spectrophotometry, EE(λ) is the erythemal effect spectrum, I(λ) is the solar intensity, and A(λ) is the absorbance measured at wavelength λ. The values of EE(λ) × I(λ) are constants and dependent on the wavelength, determined by Sayre et al. [60]. The normalized values of EE(λ) × I(λ) are presented in Table 3.

### 3.9. UVA Protection Parameters: Boots Star Rating System and Critical Wavelength

The UVA/UVB absorbance ratio (R coefficient) (Table 4) was estimated according the Boots Star Rating system [40,42] and Equation (2):

(2) R=∫320400Aλ·dλ∫320400dλ∫290320Aλ·dλ∫290320dλ

The areas under the absorption spectrum in the range of UVA (320–400 nm) and UVB (290–320 nm) were calculated using OriginPro 6.1 (Northampton, MA, USA) software.

The R values with their star ratings and protection categories [40,42] are presented in Table 4.

Critical wavelength λ_cr_ was calculated from the absorption spectra using OriginPro 6.1 (Northampton, MA, USA) software and Equation (3).

(3) $$\int_{290}^{\lambda_{cr}}A\left(\lambda \right)\cdot d\lambda =0.9\cdot \int_{290}^{400}A\left(\lambda \right)\cdot d\lambda$$

### 3.10. Statistical Analysis

The results presented for cell lines are the average of four experiments, and cytoprotective activities were subjected to statistical analysis using one-way ANOVA analysis, followed by Tukey’s multiple comparisons post hoc test performed using OriginPro 6.1 (Northampton, MA, USA) software at a significance level of *p* ≤ 0.05.

The results for the absorbance A and SPF are presented as the mean of three/four repeats and standard deviation (mean ± SD). To analyze the absorbance of pulcherrimin, non-parametric tests were used for statistical analyses of the studied parameters that did not follow a normal distribution (Shapiro–Wilk test). Differences regarding the analyzed parameters (pH) were tested using the Mann–Whitney U Test (UMW test)—a comparison of the two groups. To compare 3 or more groups, the Kruskal–Wallis test (KW test), followed by a multiple comparison test (MC test), was applied. A *p*-value < 0.05 was considered statistically significant. The UMW test, KW test, and MC test were performed using Statistica ver. 13.1 (StatSoft, Tulsa, OK, USA).

## 4. Conclusions

The aim of this research was to evaluate the photoprotective effects of yeast pulcherrimin, an iron-chelating dipeptide. Cell viability results indicate that pulcherrimin from yeast *Metschnikowia pulcherrima* exhibits both antioxidant and cytoprotective activities against human HaCaT skin keratinocytes, by reducing oxidative stress and ROS generation. Pulcherrimin was also found to induce DNA repair caused by UVC radiation. It exhibits notable antioxidant and in vitro photoprotective activities, with SPF values of 15–20 depending on the pH level of the environment, earning a 5-star rating according to the Boots Star Rating System. These parameters are in line with European Recommendation 2006/647/WE. The obtained results for HaCaT cells indicate clear statistically significant photoprotective properties of yeast pulcherrimin (*p* ≤ 0.05). The absorbance of the pulcherrimin suspension, as well as its SPF at different pH values, showed statistically significant values, when pulcherrimin was less dissolved. The obtained results concerning the photoprotective effect of yeast pulcherrimin indicate that it is worth continuing using other sources of radiation and cell lines, i.e., primary human keratinocytes and human skin histoculture. Since this is the first report on the photoprotective effects of yeast pulcherrimin, it is suggested to perform exhaustive investigations of its potential for applications in drugs and skincare products.

## 5. Patents

The results formed the basis for the Patent Application P. 447677 “The use of pulcherrimin for UV protection” to the Patent Office of the Republic of Poland.

## Figures and Tables

**Table 1:** The pulcherrimin absorbance (mean ± SD) depending on the environment pH.

λ [nm]	Absorbance A		*p* Value (UMW Test)
	pH 7	pH 10	
290	2.411 ± 0.157	1.946 ± 0.084	**0.029**
295	2.135 ± 0.138	1.738 ± 0.190	**0.030**
300	2.018 ± 0.127	1.639 ± 0.116	**0.030**
305	1.977 ± 0.121	1.600 ± 0.118	**0.030**
310	1.955 ± 0.120	1.582 ± 0.120	**0.030**
315	1.933 ± 0.115	1.554 ± 0.127	**0.030**
320	1.919 ± 0.116	1.549 ± 0.123	**0.030**
SPF *	20.00 ± 1.44 (20 *)	16.20 ± 1.36 (15 *)	**0.030**

* SPF parameter designed based on Commission Recommendation of 22 September 2006 on the efficacy of sunscreen products and the claims made relating thereto [28]. The absorbance A and SPF values represent the mean ± standard deviation (SD). Statistically significant differences (Mann–Whitney U Test) in absorbance are indicated in bold.

**Table 2:** Critical wavelength, R value, and star ratings for pulcherrimin at pH = 7 and pH = 10.

pH Level	λ_cr_ [nm]	R	Star Rating	Protection Category
7	387.99 ± 0.47	1.08 ± 0.02	*****	ultra
10	389.00 ± 0.88	1.05 ± 0.02	*****	ultra

Each critical wavelength λ_cr_ and R value represents the mean of four repetitions ± the standard deviation (SD). *****—maximum UV protection.

**Table 3:** Normalized EE(λ) × I(λ) values.

λ [nm]	EE(λ) × I(λ)
290	0.0150
295	0.0817
300	0.2874
305	0.3278
310	0.1864
315	0.0839
320	0.0180

**Table 4:** The R values and their assigned star ratings in the Boots Star Rating System.

R	Star Rating	Protection Category
0–0.2	None	none
0.21–0.41	*	minimal
0.42–0.61	**	moderate
0.62–0.81	***	good
0.82–0.91	****	superior
>0.92	*****	ultra

The number of asterisks indicates the level of protection against UV radiation, where “*” is no protection and “*****” is maximum UV protection.

## Data Availability

Data are contained within the article.

## Supplementary Materials

The following supporting information can be downloaded at: https://www.mdpi.com/article/10.3390/molecules29204873/s1, Table S1: The pulcherrimin absorbance (Mean ± SD) depending on the wavelength. Statistically significant differences according the Kruskal-Wallis (KW) test; Figure S1: The pulcherrimin absorbance (Mean ± SD) at pH = 7 depending on the wavelength; Figure S2: The pulcherrimin absorbance (Mean ± SD) at pH = 10 depending on the wavelength; Figure S3: The pulcherrimin SPF (Mean ± SD) depending on the pH.
