# Supplementary material for: Photoprotective Effects of Yeast Pulcherrimin
Source: Molecules. 2024 Oct 14;29(20):4873. doi: 10.3390/molecules29204873 (PMC11510698; doi:10.3390/molecules29204873)
Supplement: Supplementary file 1 [file molecules-29-04873-s001.zip › molecules-3154648-supplementary.pdf]

Manuscript ID: molecules-3154648

Photoprotective effects of yeast pulcherrimin

Supplementary material

Table S1. The pulcherrimin absorbance (Mean ± SD) depending on the wavelength. Statistically significant differences according the Kruskal-Wallis (KW) test.

pH	290 nm	295 nm	300 nm	305 nm	310 nm	315 nm	320 nm
7	2.411 ± 0.183	2.135 ± 0.159	2.018 ± 0.147	1.977 ± 0.141	1.955 ± 0.139	1.933 ± 0.133	1.919 ± 0.134
10	1.946 ± 0.097	1.738 ± 0.126	1.639 ± 0.135	1.600 ± 0.137	1.582 ± 0.139	1.554 ± 0.147	1.549 ± 0.142
p value (KW test)	p>0.05	p>0.05	p>0.05	p>0.05	p>0.05	p>0.05	p>0.05

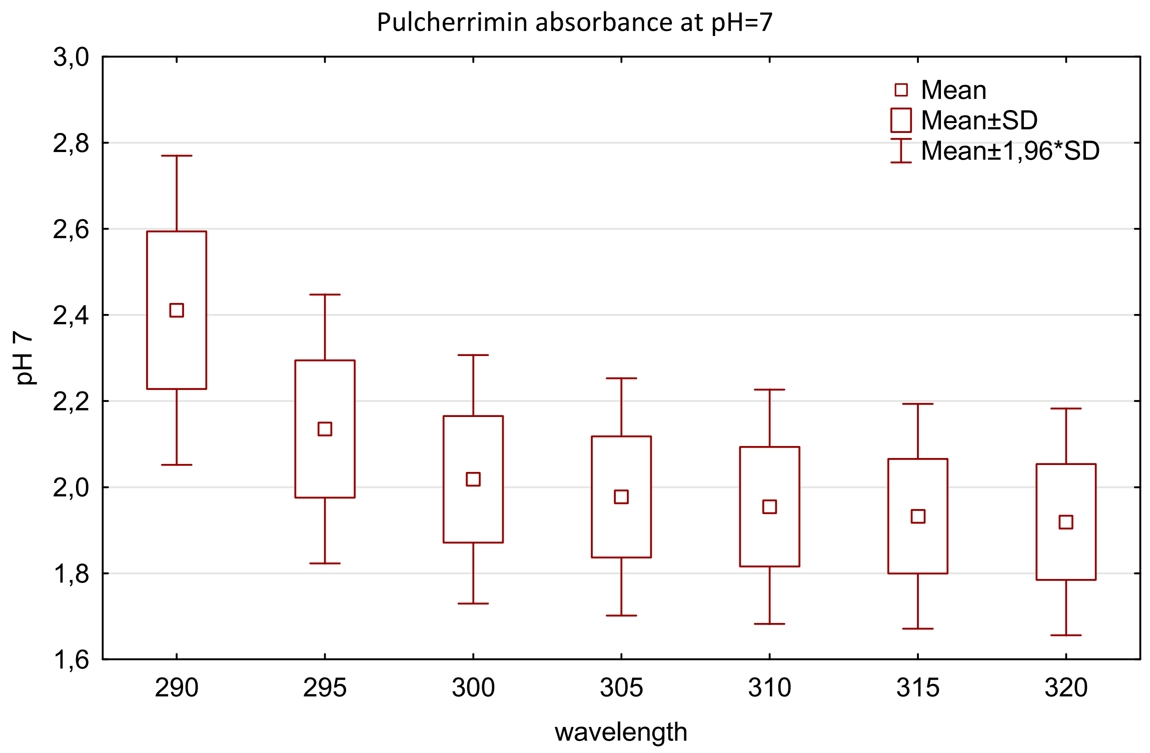

Figure S1. The pulcherrimin absorbance (Mean ± SD) at pH=7 depending on the wavelength.

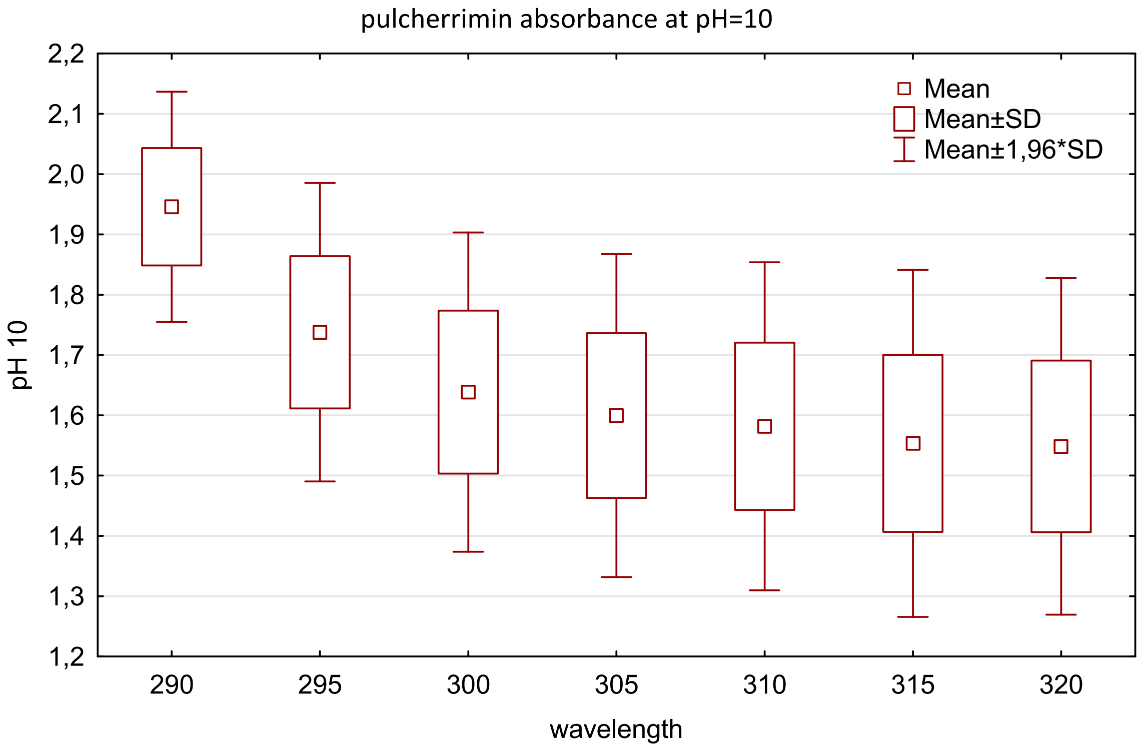

Figure S2. The pulcherrimin absorbance (Mean ± SD) at pH=10 depending on the wavelength.

The comparison of the pulcherrimin absorbance at different wavelengths (λ 290mnm, 290 nm, 300 nm, 305 nm, 310 nm, 315 nm, 320 nm) revealed no statistically significant differences (Kruskal-Wallis test, followed by a multiple comparison test, $p>0.05$) for both absorbance at pH=7 and pH=10.

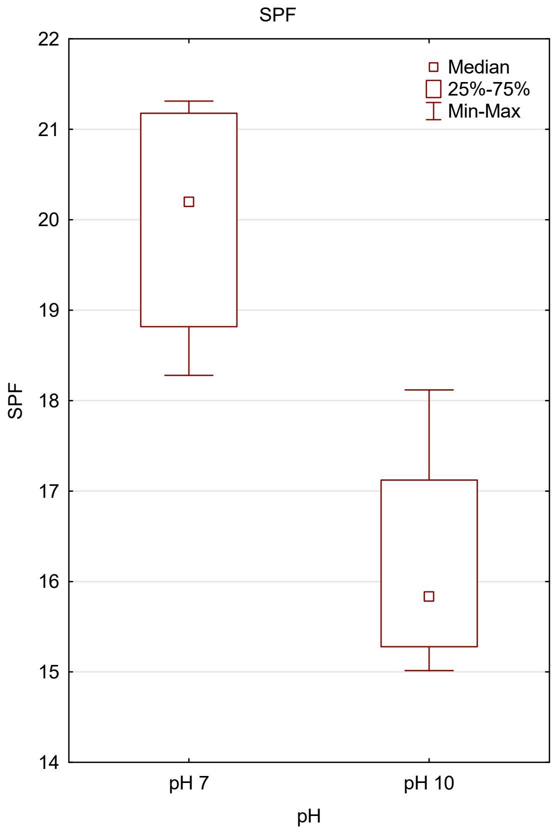

Figure S3. The pulcherrimin SPF (Mean ± SD) depending on the pH.

The comparison of the pulcherrimin SPF at pH=7 and pH=10 revealed statistically significant higher SPF in pH=7 (Mann-Whitney U Test, $p=0.030$).
